# Supplementary material for: The long non-coding RNA NEAT1 is a ΔNp63 target gene modulating epidermal differentiation
Source: Nat Commun. 2023 Jun 26;14:3795. doi: 10.1038/s41467-023-39011-5 (PMC10293300; doi:10.1038/s41467-023-39011-5)
Supplement: Supplementary file 1 — Supplementary Information [file 41467_2023_39011_MOESM1_ESM.pdf]

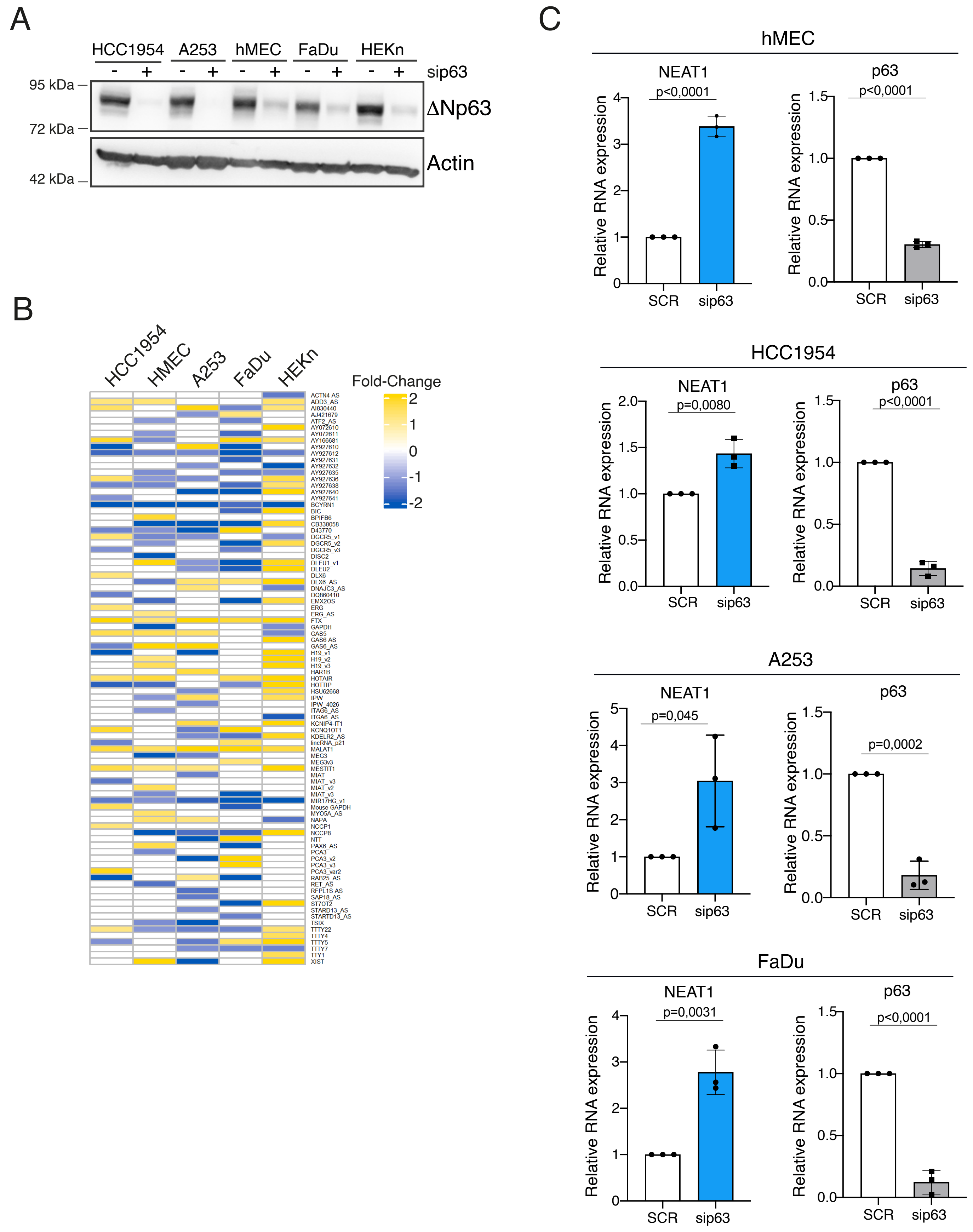

Figure S1. A) The indicated cell types were transfected with scramble (-) or siRNA oligos targeting p63 mRNA (sip63). Forty-eight hours after transfection, whole protein lysates were subjected to immunoblotting using antibodies to the indicated proteins. Representative image of two independnet experiments (n=2). B) Heatmap showing the lncRNAs expression fold change in transfected cells (|log2 fold change (FC)| > 1, adjusted P < 0.05). P= FDR (False Discovery Rate) adjusted p value calculated by Limma package from R. Color scheme: yellow (highest) blue (lowest) VSD score. C) The indicated cell types were transfected with scramble (SCR) or siRNA oligos targeting p63 mRNA (sip63). Forty-eight hours after transfection, p63 and NEAT1 RNA levels were quantified by RT-qPCR. Data shown are the mean of three (n=3) independent biological replicates ± SD. p value was calculated using two-tailed unpaired Student's t test. Source data are provided as a Source Data file.

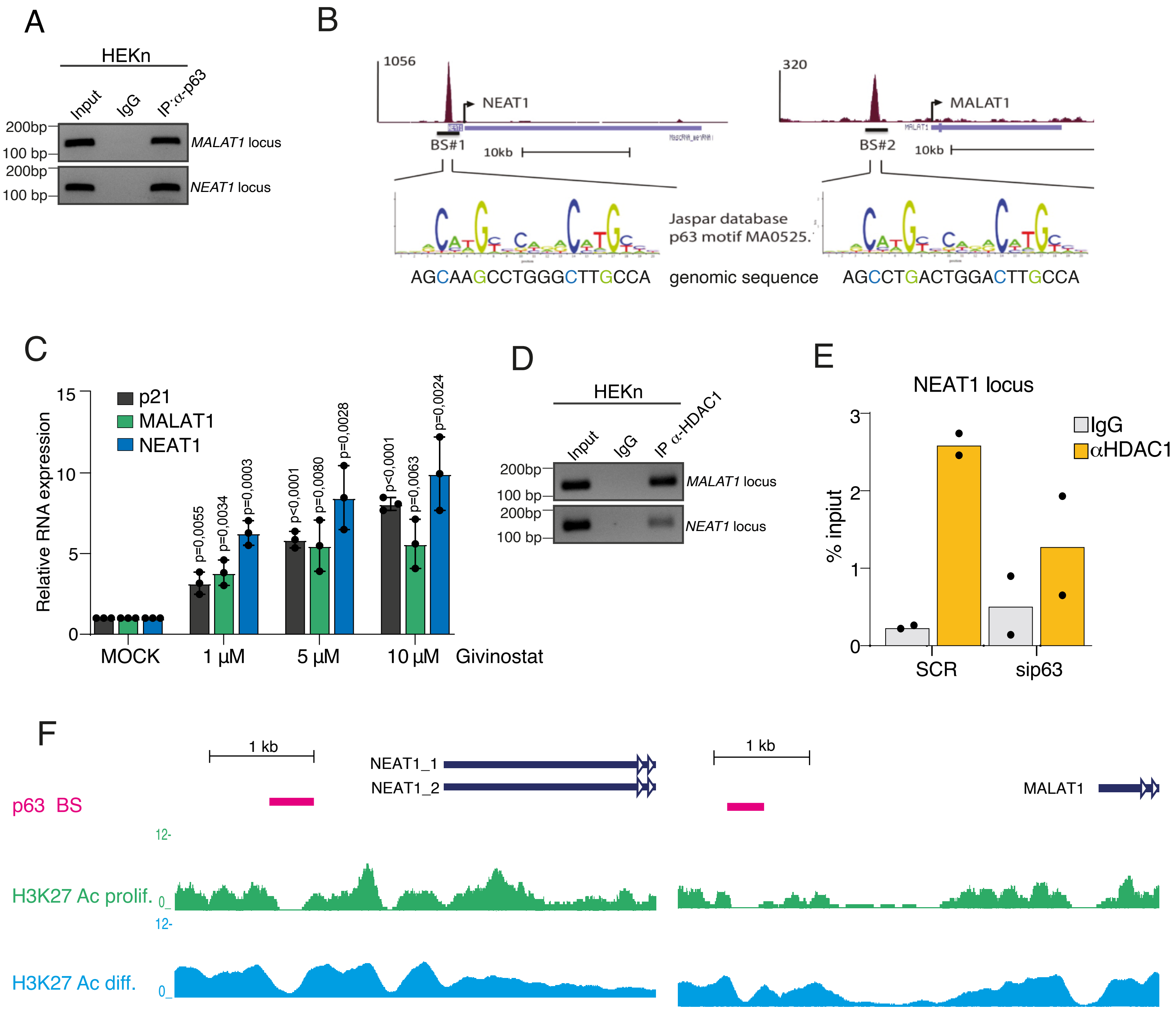

Figure S2. A) Representative agarose gel image showing the PCR amplification of endogenous ΔNp63 occupancy at the p63 DNA binding sites localized at MALAT1 and NEAT1 loci by ChIP assay. ChIP analysis was performed in human primary keratinocytes (HEKn). Similar results were obtained by analyzing two additional biological replicates (n=2) by qPCR (see Figure 1F). B) p63 DNA-binding profiles in the MALAT1 and NEAT1 genomic loci obtained in NHEK by ChIP-seq (GSM1446927). Canonical p63 DNA binding motif was extrapolated by JASPAR database. C) HEKn cells were treated for 24 hours with the indicated concentration of HDAC inhibitor Givinostat and RNA levels of the indicated genes were quantified by RT-qPCR. Data shown are the mean of three (n=3) independent biological replicates ± SD. p value was calculated using two-tailed unpaired Student's t test. D) Representative agarose gel image showing the PCR amplification of endogenous HDAC1 occupancy at the p63 DNA binding sites localized at MALAT1 and NEAT1 loci in human primary keratinocytes (HEKn) by ChIP assay. Similar results were obtained by analyzing two additional biological replicates (n=2) by qPCR (see Figure 1G). E) ChIP-qPCR showing HDAC1occupancy at MALAT1 and NEAT1 genomic loci in HEKn cells transfected with scramble (SCR) or siRNA oligo targeting p63 (sip63). Average values from n = 2 biological replicates measured using three technical replicates are plotted. F) H3K27ac profile of the promoter region of MALAT1 and NEAT1 genomic loci in proliferating and differentiating primary keratinocytes. H3K27ac profile was downloaded by the GEO dataset GSM1446919 (prolif.) and GSM1446922 (diff.). p63 ChIP-seq data were downloaded by the GEO dataset GSM1446930. Source data are provided as a Source Data file.

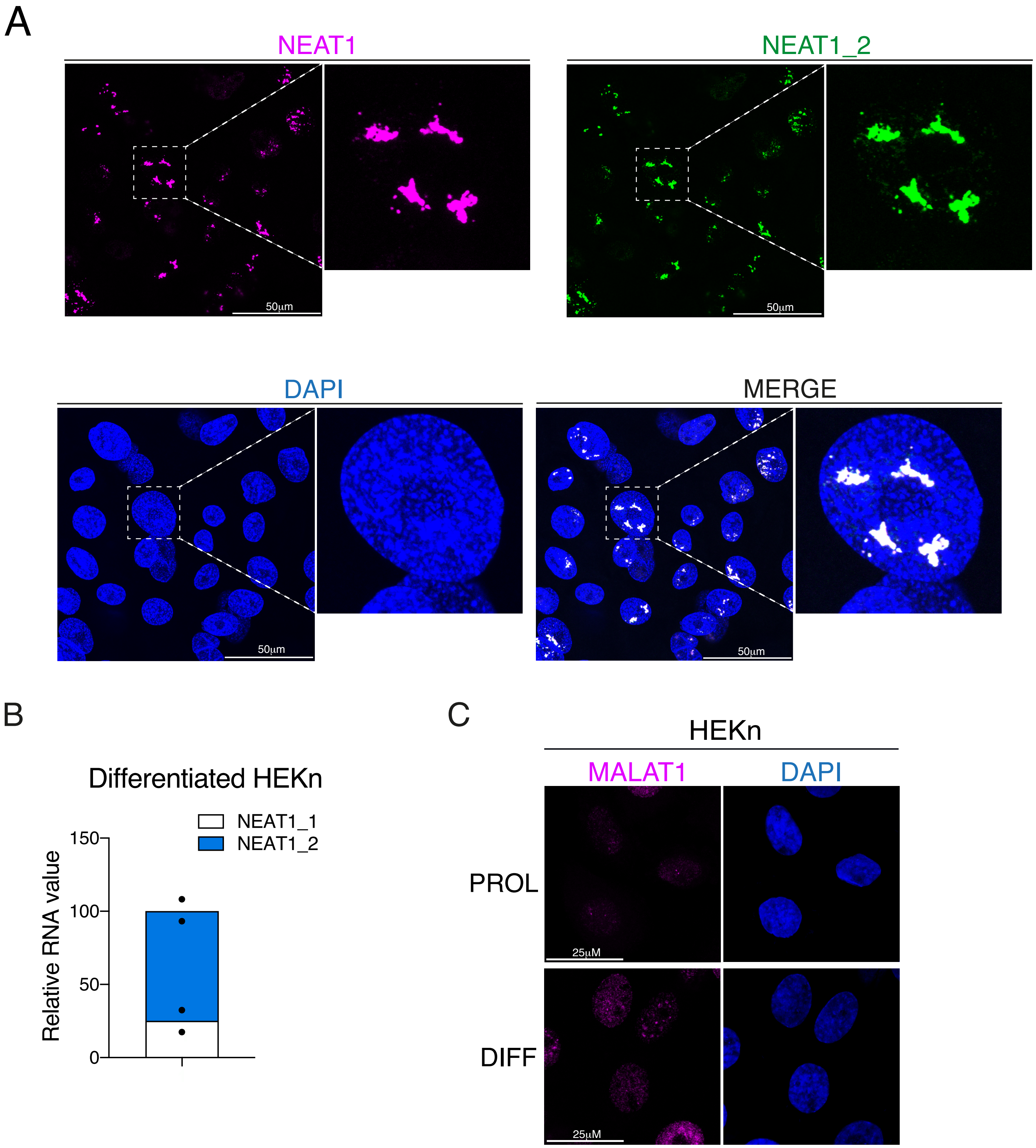

Figure S3. A) Ultraresolution analysis (LEICA Stellaris 5 Lightning LasX) of the RNA FISH of endogenous NEAT1 in differentiated keratinocytes (6 days in high calcium medium). NEAT1 was detected by utilizing a probe common to the 5' common region of NEAT1_1 and NEAT1_2 (magenta) or a NEAT1_2 specific probe (green). Nuclei were visualized by DAPI (blue) counterstaining. Scale bars, 50 µm. B) Stacked bar graph representing the ratio of NEAT1_1/ NEAT1_2 isoforms in differentiated keratinocytes (6 days in high calcium medium). Data shown are the mean of two (n=2) independent biological replicates. C) Representative confocal images of RNA FISH of endogenous MALAT1 (magenta) in proliferating (PROL) or differentiated (DIFF) HEKn cells. Nuclei were visualized by DAPI (blue) counterstaining. Scale bars, 25 µm. The experiment was repeated twice with similar results (n=2). Source data are provided as a Source Data file.

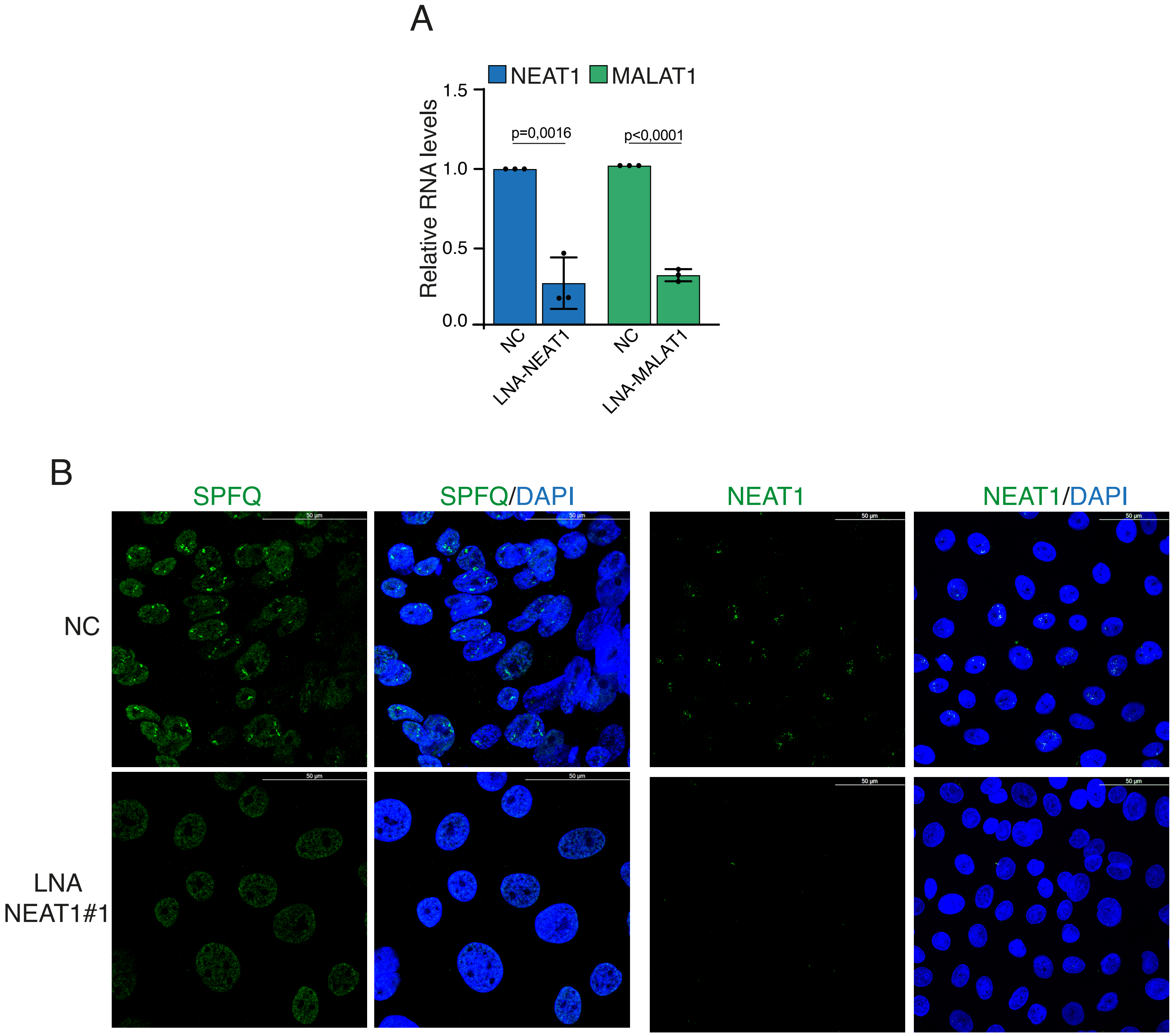

Figure S4. A) RT-qPCR analysis of NEAT1 and MALAT1 RNA levels in differentiated keratinocytes upon NEAT1 (LNA-NEAT1) or MALAT1 (LNA-MALAT1) depletion. Data shown are the mean of three (n=3) independent biological replicates ± SD. p value was calculated using two-tailed unpaired Student's t test. B) Left panel: immunostaining of SFPQ (green) in differentiated keratinocytes (4 days in high calcium medium) upon NEAT1 depletion; right panel: RNA FISH of endogenous NEAT1 (green) in differentiated keratinocytes (4 days in high calcium medium) upon NEAT1 depletion. Nuclei were visualized by DAPI (blue) counterstaining. Scale bars, 50 μm. Source data are provided as a Source Data file.

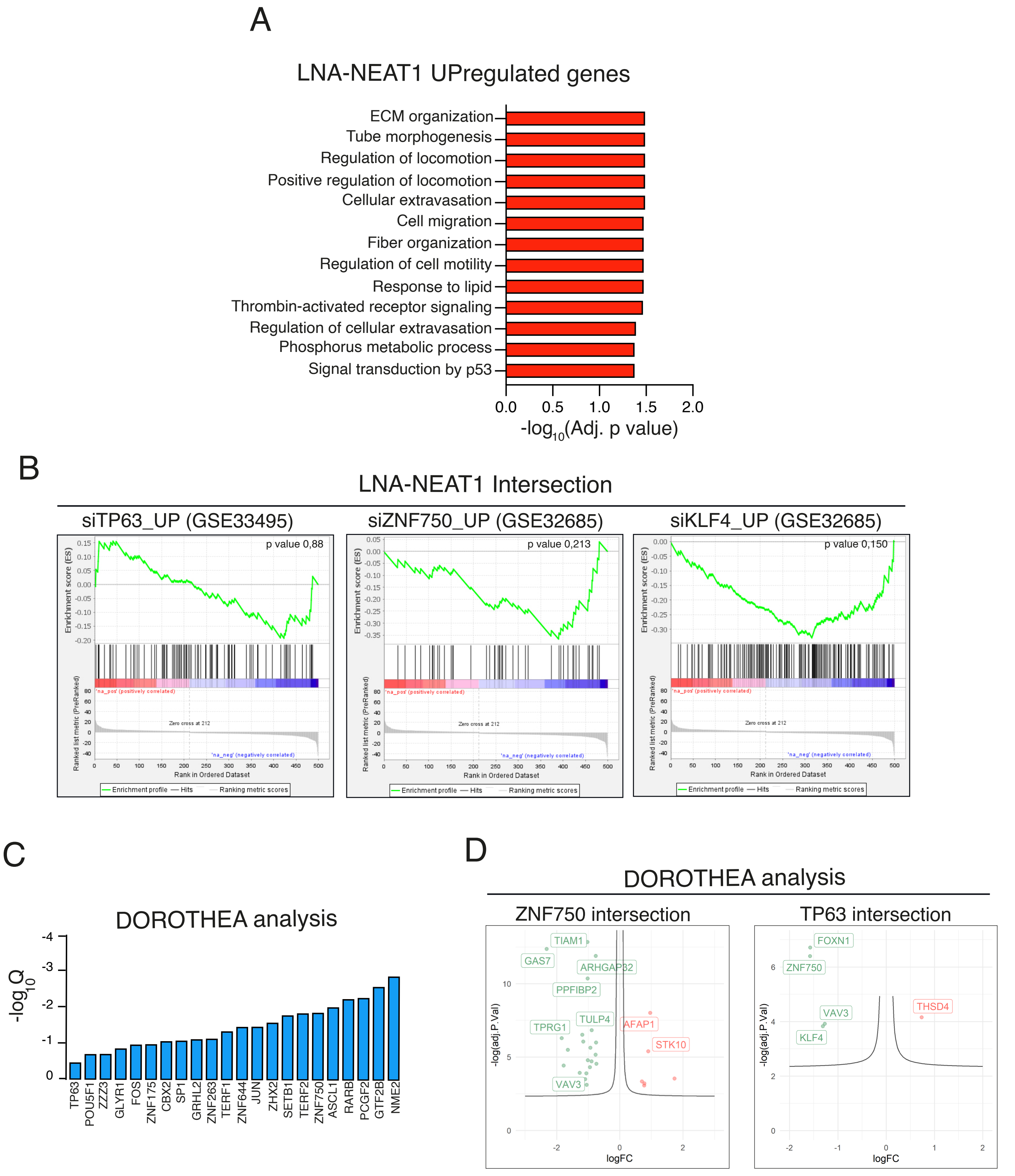

Figure S5. A) Barplot showing the top gene ontology (GO) terms for Biological Process of the upregulated genes upon LNA-NEAT1 transfection of differentiated HEKn cells. GO terms were ordered by FDR (False Discovery Rate) adjusted p value calculated by ShinyGO version 0.76. B) GSEA of LNA-NEAT1 downregulated genes set against three keratinocytes differentiation signatures of upregulated genes resulted by p63 (GSE33495), ZNF750 or KLF4 (GSE32685) depletion in human keratinocytes. C) Transcription factors (TFs) signature enrichment of NEAT1-downregulated genes by DoRothEA analysis. D) ZNF750 and Tp63 transcriptional targets enrichment in NEAT1-downregulated genes by DoRothEA analysis. P= FDR (False Discovery Rate) adjusted p value caluclated by R software.

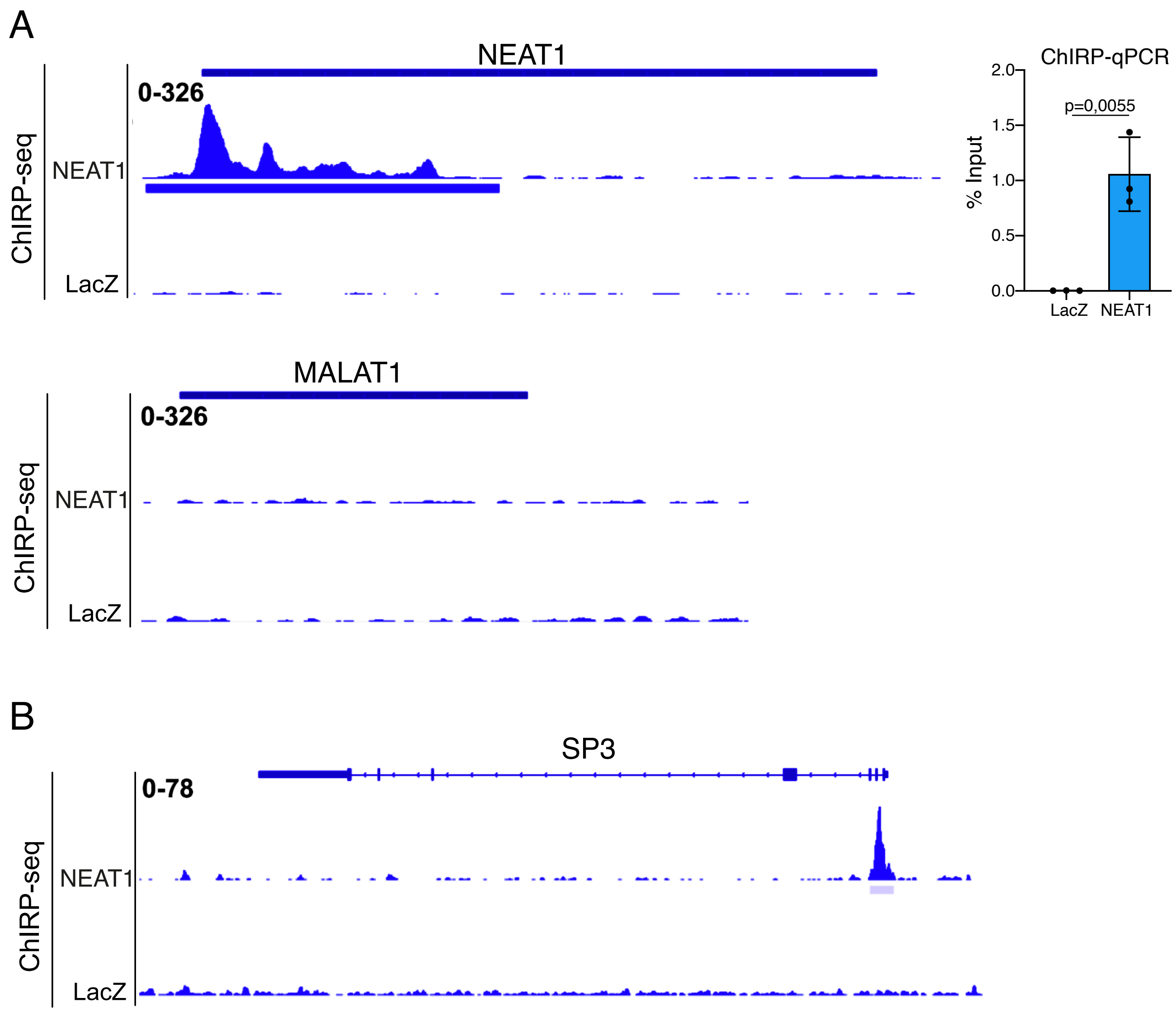

Figure S6. A) LacZ or antisense NEAT1 ChIRP enrichment over NEAT1-MALAT1 in differentiated keratinocytes. NEAT1 ChIRP enrichment over the NEAT1 locus has been also confirmed by qPCR (right panel). Data shown are the mean of three (n=3) independent biological replicates ± SD. p value was calculated using two-tailed unpaired Student's t test. B) LacZ or antisense NEAT1 ChIRP enrichment over SP3 loci in differentiated keratinocytes. Source data are provided as a Source Data file.

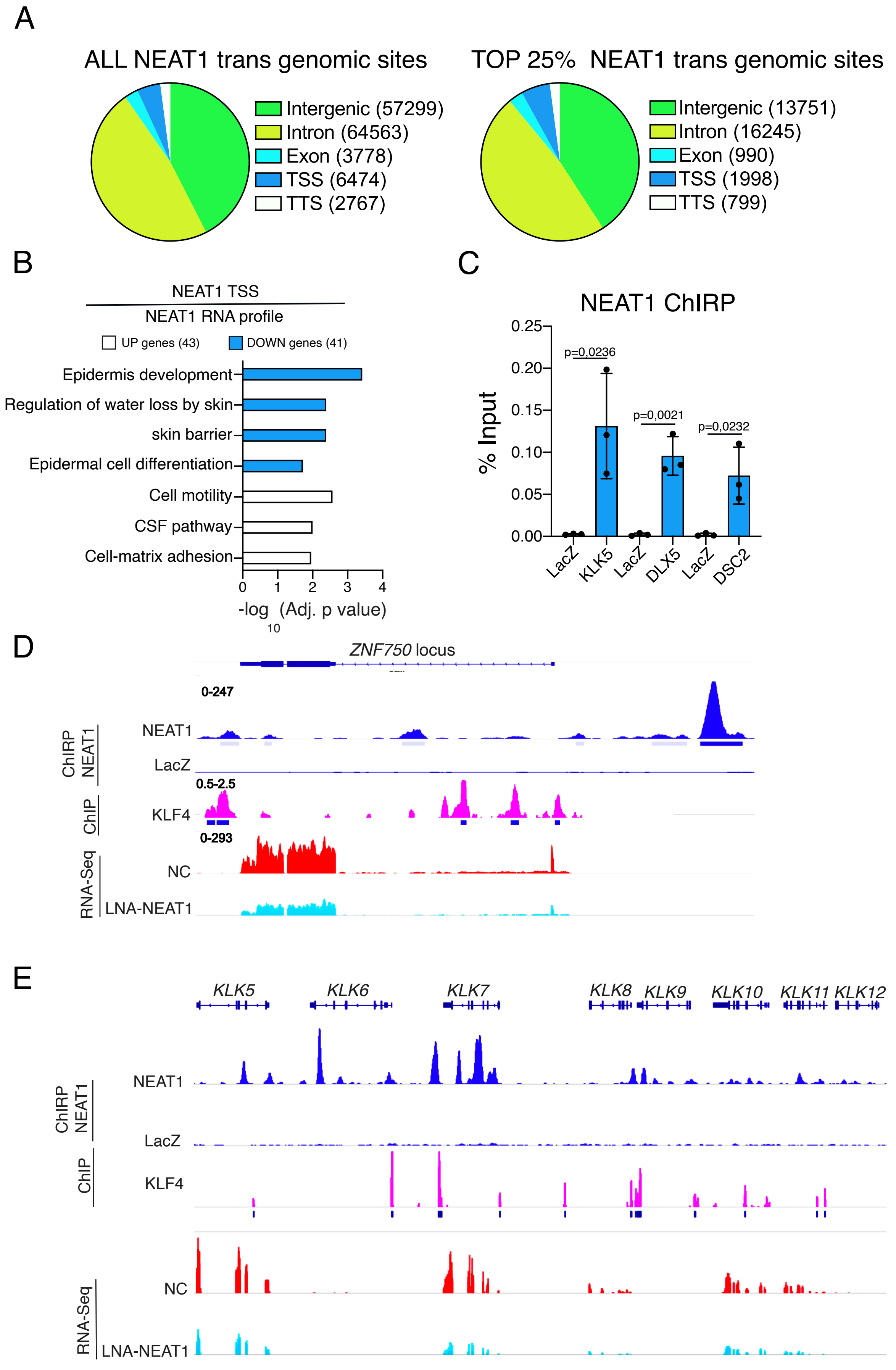

Figure S7. A) Genomic distribution of NEAT1 binding sites in differentiated keratinocytes. B) Barplot showing the top gene ontology (GO) terms for Biological Process of the intersection analysis between NEAT1 binding sites over TSSs and NEAT1 dependent RNA profile (see Figure 3) in differentiated keratinocytes, ordered by FDR (False Discovery Rate) adjusted p value calculated by ShinyGO version 0.76. C) qPCR analysis of the NEAT1 ChIRP enrichment over the indicated epidermal genes loci. Data shown are the mean of three (n=3) independent biological replicates ± SD. p value was calculated using two-tailed unpaired Student's t test. D-E) RNA-seq reads in control (NC) and NEAT1 depleted keratinocytes (LNA-NEAT1) together with NEAT1 ChIRP enrichment and KLF4 genomic occupancy (GSE57702) over the indicated epidermal genes in differentiated keratinocytes. Source data are provided as a Source Data file.

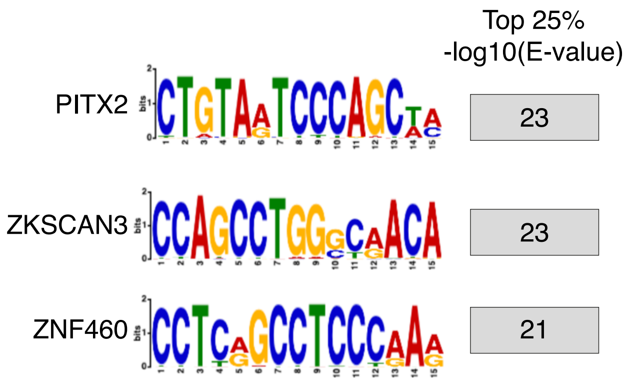

Figure S8. Top three NEAT1 DNA consensus motifs enriched in the top 25% NEAT1 trans genomic sites. DNA consensus motifs were identified by using the MEME algorithm.

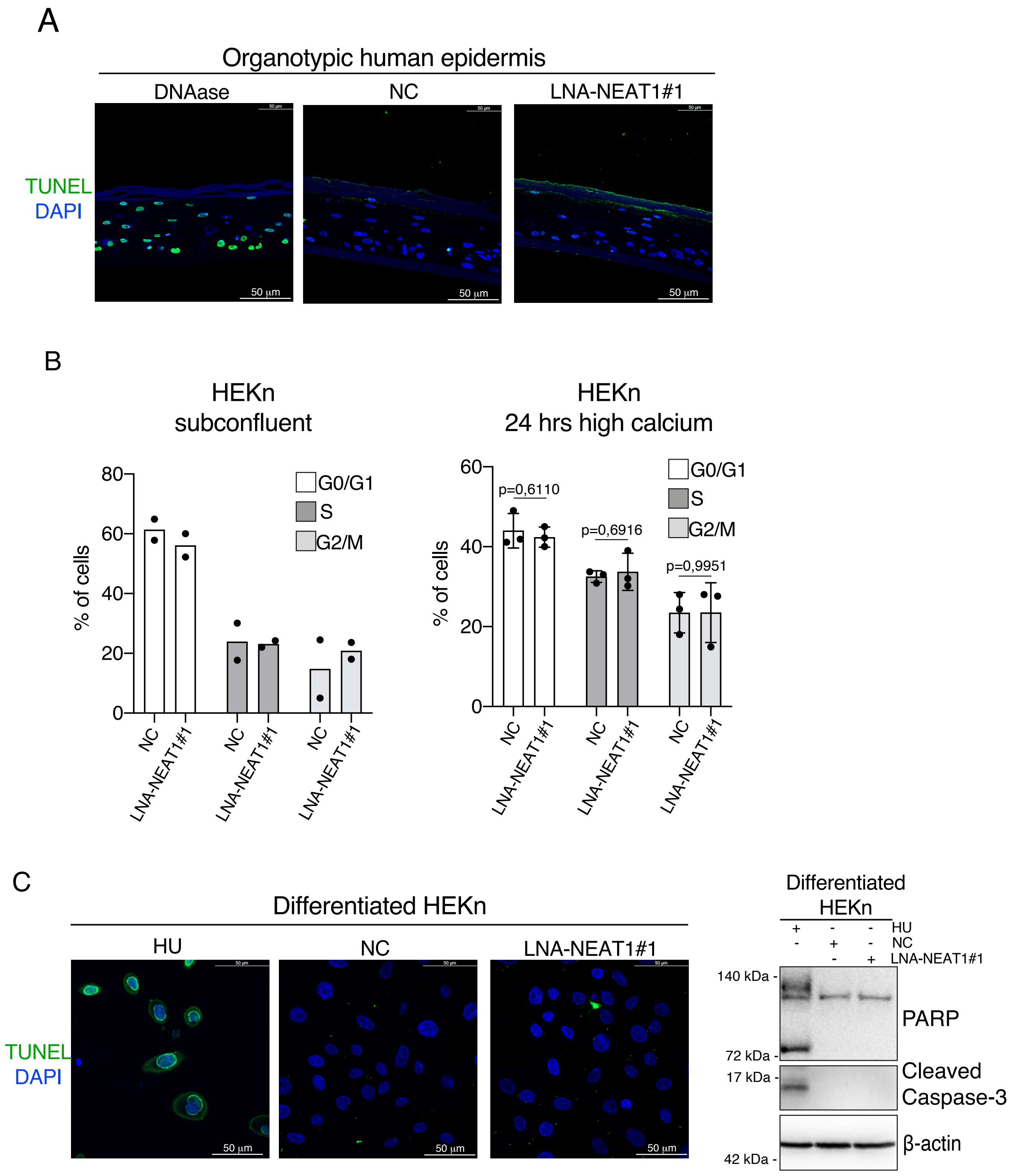

Figure S9. A) TUNEL (green) assay in control (NC) and NEAT1-depleted epidermis (LNA-NEAT1). As control, control sample was treated with DNAse. DAPI (blue) was used to visualize nuclei. The experiment was repeated twice with similar results (n=2). Scale bars, 50 μm. B) Stacked bar graph representing the cell-cycle distribution of PI/Edu staining in control (NC) and NEAT1-depleted (LNA-NEAT1#1) primary human keratinocytes (HEKn) at subconfluent condition or after 24 hours in high calcium medium. Data shown are the average of n=2 independent biological replicates (subconfluent condition) or n=3 independent biological replicates ± SD (24 hrs high calcium). p value was calculated using Multiple t-test unpaired C) TUNEL assay in in control (NC) and NEAT1-depleted (LNA-NEAT1) differentiated keratinocytes (3 days in high calcium medium). As control, control sample was treated with 1,5 mM HU for 20 hours. DAPI (blue) was used to visualize nuclei. Scale bars, 50 μm. In parallel, the protein levels of the indicated apoptotic amrkers were analyzed by IB. The experiment was repeated twice with similar results (n=2). Source data are provided as a Source Data file.

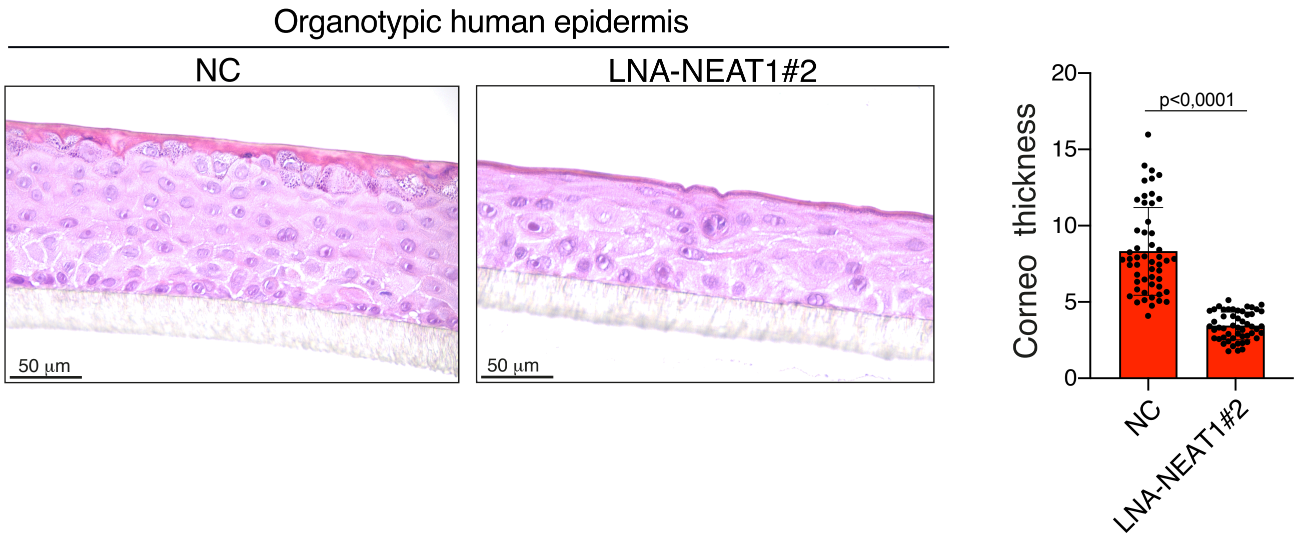

Figure S10. Representative image of H&E staining (left panel) and quantification of stratum corneum thickness (right panel) in control (NC) and NEAT1-depleted (LNA-NEAT1#2) organotypic human epidermis. The quantification of stratum corneum thickness is shown as the mean of n=52 measurements ± SD. p value was calculated using two-tailed unpaired Student's t test. Scale bars, 50 μm. Source data are provided as a Source Data file.

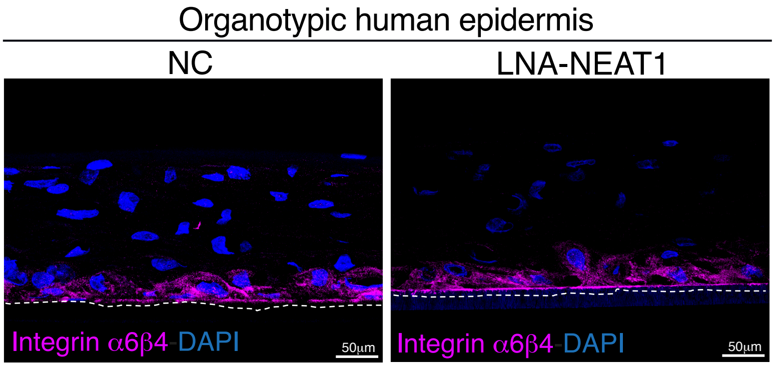

Figure S11. Representative confocal image of integrin α6β4 localization in control (NC) and NEAT1-depleted (LNA-NEAT1) organotypic skin cultures. DAPI (blue) was used to visualize nuclei. Dotted lines underline the keratinocyte-fibroblast border. The experiment was repeated twice with similar results (n=2). Scale bars, 50 µm

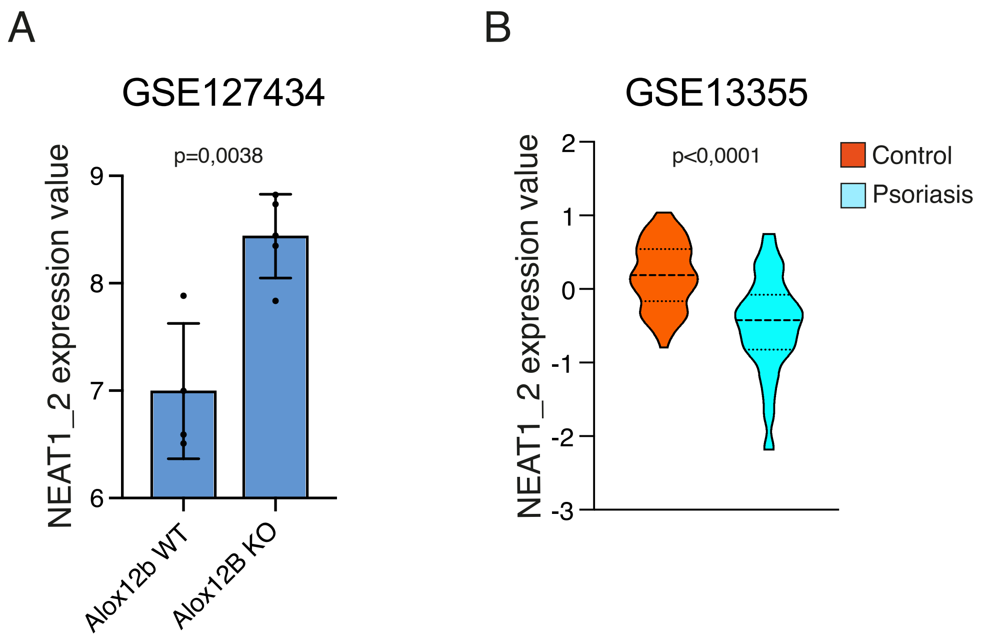

Figure S12. Analysis of NEAT1_2 isoform expression in Alox12b Knock-out (KO) (GSE127434) and psoriasis dataset (GSE13355). In the Alox12b KO dataset NEAT1_2 expression value (DNA probe 227062_at) is shown as the mean ± SD of 4 (n=4, normal skin) and 5 (n=5 Ichthyosis lesions) samples. In the psoriasis dataset, NEAT1_2 expression value (DNA probe 227062_at) is shown as the mean ± SD of 64 (n=64, normal skin) and 58 (n=58, psoriatic lesions) samples. p value was calculated using two-tailed unpaired Student's t test. Source data are provided as a Source Data file.

Figure S1A

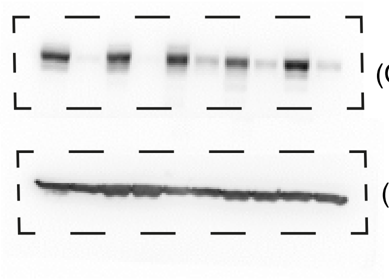

anti p63-α
(Cell Signaling #D2K8X)

anti β-actin
(Sigma-Aldrich #AC-15)

Figure S2A

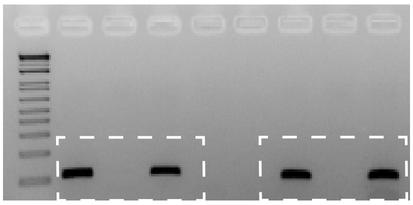

ChIP anti-p63-α
(Cell Signaling #D2K8X)

hMALAT1
promoter

hNEAT1
promoter

Figure S2D

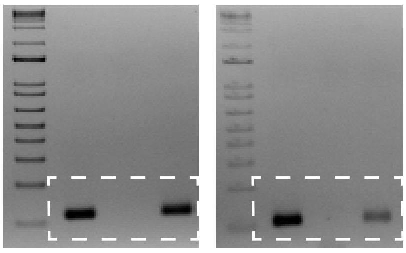

ChIP anti-HDAC1
(Abcam #Ab19845)

hMALAT1
promoter

hNEAT1
promoter

Figure S9C

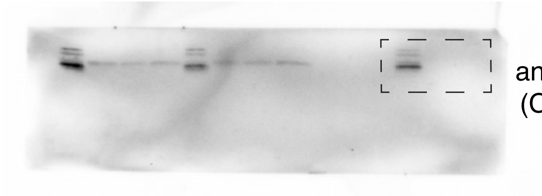

anti-cleaved-Caspases 3
(Cell Signaling #5A-1E)

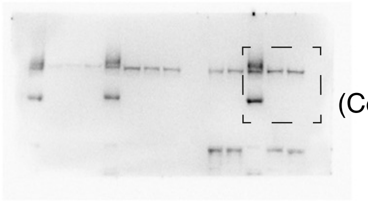

anti PARP
(Cell Signaling #9542)

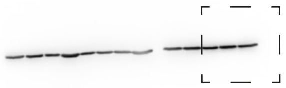

Anti β-actin
(Sigma-Aldrich #AC-15)

Figure S13. Uncropped images related to the blots/gels shown in the Supplementary Figures.

Table S1. Deregulated lncRNAs upon p63 silencing

Common UPregulated lncRNAs upon p63 silencing FCH_sip63/SCR					
	HEKn	A253	FaDu	HMEC	HCC1954
MALAT1	1,77	2,2	2,9	1,51	1,79
FTX	2,2	2,7	1,83	1,35	2,17
Common DOWNregulated lncRNAs upon p63 silencing FCH_sip63/SCR					
	HEKn	A253	FaDu	HMEC	HCC1954
BCYRN1	-5,78	-5,47	-1,71	-2,27	-2,76
MIR17HK	-2,89	-1,85	-4,26	-1,24	-1,55
AY827612	-1,5	-1,37	-3,23	-1,42	-1,77

Table S2. siRNA and LNA GapmeR oligos list (sequence 5'-3')

siRNA p63	CAGGUUGGCACUGAAUUCA
siRNA ΔNp63	GAAGAAAGGACAGCAGCATTG
LNA-NEAT1#1	TAAGCACTTTGGAAAG
LNA-NEAT1#2	ATCGACCAAACACAGA
LNA-MALAT1	CGTTAACTAGGCTTTA

Table S3. qPCR primer list

**Primer name**	**Sequence 5→3**
hNEAT1 for	TGGCTAGCTCAGGGCTTCAG
hNEAT1 rev	TCTCCTTGCCAAGCTTCCTTC
hNEAT1_2 for	GGCCAGAGCTTTGTTGCTTC
hNEAT1_2 rev	GGTGCGGGCACTTACTTACT
hMALAT1 for	GACGGAGGTTGAGATGAAGC
hMALAT1 rev	ATTCGGGGCTCTGTAGTCCT
hZNF750 for	AGCTCGCCTGAGTGTGAC
hZNF750 rev	TGCAGACTCTGGCCTGTA
hΔNp63 for	GAAGAAAGGACAGCAGCATTG
hΔNp63 rev	GGGACTGGTGGACGAGGAG

hp21 for	GTCACTGTCTTGTACCCTTGTG
hp21 rev	CGGCGTTTGGAGTGGTAGAAA
hKRT10 for	AGGAGGAGTGTCATCCCTAAG
hKRT10 rev	AAGCTGCCTCCATAACTCCC
hKRT1 for	GGTGAAGTCTCGAGAAAGGGA
hKRT1 rev	TGGTCCACTCTCCTTCGGA
hTBP for	CTGACAGGTAAGGAGGACGC
hTBP rev	AGTTACCTGACCTCTCCCCC
hNEAT1 ChIP for	GAAACAGGGCTAAGCAGGCG
hNEAT1 ChIP rev	GCTTCTCGGAAAACTGGTGAC
hMALAT1 ChIP for	GGCCGTAGATGACGGATCTC
hMALAT1 ChIP rev	GCCGGAAACCTCCAGAGAAT
hNEAT1 ChIRP/ChIP for	GCTTGGCAAGGAGACTAGGT
hNEAT1 ChIRP/ChIP rev	GGGACCCTGCGGATATTTTC
hKLK5 ChIRP/ChIP for	ATAACCACCCACTGCTCCCT
hKLK5 ChIRP/ChIP rev	GAGCTATTGCTAAGGCCCGA
hDLX5 ChIRP/ChIP for	AGGGGAGAGACGCTAGTTCG
hDLX5 ChIRP/ChIP rev	GTTTCTCGGGCCAGCATTTT
hDSC2 ChIRP/ChIP for	CCCCGTTCCCCTAGTTTTCC
hDSC2 ChIRP/ChIP rev	CTCGCGGTGAGTGTAGCC
