# Supplementary material for: The long non-coding RNA NEAT1 is a ΔNp63 target gene modulating epidermal differentiation
Source: Nat Commun. 2023 Jun 26;14:3795. doi: 10.1038/s41467-023-39011-5 (PMC10293300; doi:10.1038/s41467-023-39011-5)
Supplement: Supplementary file 3 — Reporting Summary [file 41467_2023_39011_MOESM3_ESM.pdf]

nature portfolio

Corresponding author(s): Angelo Peschiaroli

Last updated by author(s): May 17th, 2023

Reporting Summary

Nature Portfolio wishes to improve the reproducibility of the work that we publish. This form provides structure for consistency and transparency in reporting. For further information on Nature Portfolio policies, see our Editorial Policies and the Editorial Policy Checklist.

Statistics

For all statistical analyses, confirm that the following items are present in the figure legend, table legend, main text, or Methods section.

n/a	Confirmed	
☐	☒	The exact sample size (*n*) for each experimental group/condition, given as a discrete number and unit of measurement
☐	☒	A statement on whether measurements were taken from distinct samples or whether the same sample was measured repeatedly
☐	☒	The statistical test(s) used AND whether they are one- or two-sided<br>*Only common tests should be described solely by name; describe more complex techniques in the Methods section.*
☒	☐	A description of all covariates tested
☒	☐	A description of any assumptions or corrections, such as tests of normality and adjustment for multiple comparisons
☐	☒	A full description of the statistical parameters including central tendency (e.g. means) or other basic estimates (e.g. regression coefficient) AND variation (e.g. standard deviation) or associated estimates of uncertainty (e.g. confidence intervals)
☐	☒	For null hypothesis testing, the test statistic (e.g. *F*, *t*, *r*) with confidence intervals, effect sizes, degrees of freedom and *P* value noted<br>*Give P values as exact values whenever suitable.*
☒	☐	For Bayesian analysis, information on the choice of priors and Markov chain Monte Carlo settings
☒	☐	For hierarchical and complex designs, identification of the appropriate level for tests and full reporting of outcomes
☒	☐	Estimates of effect sizes (e.g. Cohen's *d*, Pearson's *r*), indicating how they were calculated

Our web collection on statistics for biologists contains articles on many of the points above.

Software and code

Policy information about availability of computer code

Data collection	Images: Leica Stellaris 8 , NIKON A1 and Leica DM6<br>Western blotting: Uvitec Alliance Q9<br>qPCR: Applied Biosystem QuanStudio 5 Real-Time PCR system
Data analysis	Leica Application SuIte x (LASX)<br>NIKON NIS Elements software A1<br>UVITEC Nine Alliance software<br>Graph Pad Prism 8.0<br>IGV_2.11.2<br>Applied Biosystem QuanStudio Design and Analysis software<br>Image J software 1.53a<br>VENN (http://www.interactivenn.net)<br>ChIRP-seq data were analyzed with “chipseq” version 1.2.2 pipeline included into the nf-core platform (https://nf-co.re/chipseq) with default parameters (Ewels et al 2020) (doi: 10.5281/zenodo.3240506);<br>ShinyGO version 0.76 (Ge et al 2020);<br>ComplexHeatmap R package (Gu et al 2016);<br>DoRothEA software (Garcia-Alonso et al., 2019);<br>GREAT tool (Regions Enrichment of Annotations Tool) (Mclean et al., 2010);<br>RNA-seq data were analyzed with “rnaseq” version 3.3 pipeline included into the nf-core platform (https://nfco.re/rnaseq) with default parameters (Ewels et al., 2020) (doi:10.5281/zenodo.5550247);<br>DESeq2 package (Love et al., 2014);

IPA tool (Kramer et al, 2014);
Limma library;
Regions Enrichment of Annotations Tool (GREAT) (McLean et al, 2010);
MEME algorithm (Bailey et al, 2015);
EnhancedVolcano R package;
Intervene software (Khan & Mathelier, 2017);
IPA tools (Kramer et al., 2014);

For manuscripts utilizing custom algorithms or software that are central to the research but not yet described in published literature, software must be made available to editors and reviewers. We strongly encourage code deposition in a community repository (e.g. GitHub). See the Nature Portfolio guidelines for submitting code & software for further information.

Data

Policy information about availability of data

All manuscripts must include a data availability statement. This statement should provide the following information, where applicable:

- Accession codes, unique identifiers, or web links for publicly available datasets
- A description of any restrictions on data availability
- For clinical datasets or third party data, please ensure that the statement adheres to our policy

All sequencing data that support the findings of this study have been deposited in the National Center for Biotechnology Information Gene Expression Omnibus (GEO) under the Series accession number GSE205961. This Series includes the access to RNA-seq data (GSE205960) ChIRP-seq data (GSE205959) and lncRNAs microarray (GSE232654). Public ChIP-seq was downloaded from GEO database under the accession number GSM1446927. Public RNA-seq data were downloaded from GEO database under the accession numbers GSM1446880 and GSM1446883. Public lamellas Ichthyosis and psoriasis microarray data were downloaded from GEO database under the accession numbers GSE108640 and GSE13555, respectively. Public ALOX12B knock-out mice microarray data were downloaded from GEO database under the accession number (GSE127435). Source data are provided with this paper.

Human research participants

Policy information about studies involving human research participants and Sex and Gender in Research.

Reporting on sex and gender	N/A
Population characteristics	N/A
Recruitment	N/A
Ethics oversight	N/A

Note that full information on the approval of the study protocol must also be provided in the manuscript.

Field-specific reporting

Please select the one below that is the best fit for your research. If you are not sure, read the appropriate sections before making your selection.

☒ Life sciences ☐ Behavioural & social sciences ☐ Ecological, evolutionary & environmental sciences

For a reference copy of the document with all sections, see nature.com/documents/nr-reporting-summary-flat.pdf

Life sciences study design

All studies must disclose on these points even when the disclosure is negative.

Sample size	No statistical methods were used to determine the sample size. Sample sizes were chose as large as possible to yield high statistical significance.
Data exclusions	No samples were excluded.
Replication	All experiments were repeated to assure reproducibility (the number of biological replicates per experiment varied and it is mentioned in the figure legends). All attempts for replication shows similar results.
Randomization	For drug treatments, samples were allocated into each group randomly. In each imaging session, samples were acquired selecting randomly approximately the same number of images for each condition in order to control for any variance. There are no other experiments that require randomization and therefore no randomization was performed.
Blinding	For paraspeckles and ki67 positive cells counts, and corneo thickness measurement, the researcher was single-blinded during imaging acquisition and analysis. For others experiments, no blinding was used as there are no handling steps that are realistically subject to researcher bias.

Reporting for specific materials, systems and methods

We require information from authors about some types of materials, experimental systems and methods used in many studies. Here, indicate whether each material, system or method listed is relevant to your study. If you are not sure if a list item applies to your research, read the appropriate section before selecting a response.

Materials & experimental systems

n/a	Involved in the study
☐	☒ Antibodies
☐	☒ Eukaryotic cell lines
☒	☐ Palaeontology and archaeology
☒	☐ Animals and other organisms
☒	☐ Clinical data
☒	☐ Dual use research of concern

Methods

n/a	Involved in the study
☒	☐ ChIP-seq
☒	☐ Flow cytometry
☒	☐ MRI-based neuroimaging

Antibodies

Antibodies used

Anti-p63-alpha, clone D2K8X, (Cell Signaling, cat. numb. mAb #13109). Specificity: Human. Validated for WB, IP, IHC, ChIP.
Anti-p63 (mouse monoclonal , Abcam, cat. numb. AB735). Specificity: Human. Validated for IHC
Anti-KRT1 (Biolegend, cat. numb. PRB-149P). Specificity: Human. Validated for WB.
Anti-KRT10 (Covance, cat. numb. PRB-159P). Specificity: Human, mouse, canin. Validated for WB , IHC.
Anti-ZNF750 (Sigma-Aldrich, Prestige Antibody, cat. numb. HPA023012). Specificity: Human. Validated for IHC.
Anti-beta-actin (Sigma-Aldrich , cat,. numb. AC-15). Specificity: Human, rat, mouse. Validated for WB, IF, IF and E:lisa
Anti-HDAC1 (abcam, cat. numb. ab19845). Specificity: Human, mouse, rat. Validated for WB, IHC, ChIP.
Anti-Histone H3 (acetyl K9 + K14 + K18 + K23 + K27), Abcam, cat. numb. ab47915). Specificity: Human. Validated for WB, ChIP
Anti-Loricrin (Covance cat. numb. PRB-145P). Specificity: Human, mouse. Validated for WB, IHC.
Anti-SFPQ (rabbit Sigma-Aldrich PLA0181). Specificity: Human. Validated for IHC, IP, WB.
Anti-Histone H3 (tri methyl K4) (Abcam cat.numb ab8580). Specificity: Human, cow. Validated for WB, ChIP, IHC, IF.
Anti-KLF4 (RD Systems cat. numb. AF3640). Specificity: Human. Validated for WB, IF, IHC, ChIP.
Anti-BRG1 (Bethyl Laboratories cat.numb. A300-813A). Specificity: Human. Validated for WB, IP, IHC.
Anti-PARP (rabbit polyclonal Cell signaling cat. numb. 9542). Specificity: Human. Validated for WB.
Anti-Laminin (rabbit Sigma-Aldrich cat.num. L9393). Specificity: Human. Validated for IHC.
Anti-integrin a6b4 (mouse BD cat. num. 611232). Specificity: Human. Validated for WB, IF.
Anti-Ki67 (rabbit Cell Signalin cat.numb. D3B5). Specificity: Human, mouse, rat. Validated for IF, IHC.
Anti-rabbit 488 (goat LifeTechnologies, A11034).
Anti-mouse 488 (goat LifeTechnologies, A28175).
Anti-mouse 568 (goat Life Technologies, A11019).
Horsedish cojugated antibody anti mouse Igc (BioRad cat. num. 170-5047).
Horsedish cojugated antibody anti rabbit Igc (BioRad cat. num. 170-6515).
rabbit IgG and mouse IgG (Invitrogen, MAGnify Chromatin Immunoprecipitation System, 492024).

Validation

See manufacturer's information above. Anti-ZNF750 antibody has been validated for WB in Gatti et al., PNAS 2022.

Eukaryotic cell lines

Policy information about cell lines and Sex and Gender in Research

Cell line source(s)

A253 (ATCC HTB-41), FaDu (ATCC HTP-43), HCC1954 (ATCC CRL-2338) and hMEC (ATCC PCS-600-010) were purchased by ATCC. Human primary keratinocytes (HEKn) was purcased by Life Techonologies (Cat. numb. C0015C).

Authentication

No authentication was performed.

Mycoplasma contamination

Cell lines were tested negative for mycoplasma contamination using Mycoplasma detection kit MycoAlert kit (Lonza cat no LT07-218).

Commonly misidentified lines
(See ICLAC register)

No commonly misidentified cell lines were used.
